# The applicability of the digit wrinkle scan to quantify sympathetic nerve function

**DOI:** 10.1016/j.cnp.2022.03.005

**Published:** 2022-03-28

**Authors:** Maurice Sopacua, Carla M.L. Gorissen-Brouwers, Bianca T.A. de Greef, Isis B.T. Joosten, Catharina G. Faber, Ingemar S.J. Merkies, Janneke G.J. Hoeijmakers

**Affiliations:** a Department of Neurology, School of Mental Health and Neuroscience, Maastricht University Medical Center+, Maastricht, The Netherlands; b Department of Rehabilitation Medicine, Libra Revalidatie & Audiologie, Eindhoven, The Netherlands; c Department of Neurology, Curaçao Medical Center, Willemstad, Curaçao

**Keywords:** 5-point scale, Digit wrinkle scan, Small fiber neuropathy, Autonomic function, Digit vasoconstriction, Inter-observer reliability

## Abstract

**Objective:**

Stimulated skin wrinkling test (SSW) has been launched as a non-invasive diagnostic procedure. However, no normative age dependent values have been reported that can be applied in clinical practice. The objectives of the study were to (1) collect age-dependent normative values according to the 5-point scale assessment for the SSW, to (2) determine reliability scores for the obtained norm values, and to (3) introduce a new digital method for SSW assessment, the Digit Wrinkle Scan© (DWS©) for detection of wrinkles in a more quantitative manner.

**Methods:**

Firstly, 82 healthy participants were included, divided in 5 age groups. The participants underwent SSW using lidocaine and prilocaine topical cream. Secondly, 35 healthy participants were included to test whether the DWS© could be a novel manner to assess the grade of wrinkling quantitatively. We determined the inter-observer reliability of both methods. Also, the intra-observer reliability was calculated for the DWS©.

**Results:**

We found a decrease in normative values over age. The inter-observer reliability of assessment by the 5-point scale method was moderate after SSW (Cohen’s k: 0.53). Results of the DWS© indicate that total wrinkle length per mm^2^ showed moderate to good agreement for the 4th and 5th digits after SSW, and a low agreement for the other digits.

**Conclusions:**

Age-dependent normative values were obtained according to the 5-point scale, but its clinical application is doubtful since we found a moderate inter-observer reliability. We introduced the DWS© as a possible new method in order to quantify the grade of wrinkling.

**Significance:**

We found unsatisfactory reliability scores, which hampers its usefulness for clinical practice.

## Highlights

• Normative values for stimulated skin wrinkling are age-dependent.
• Stimulated skin wrinkling has never been evaluated quantitatively.
• The clinical application of the stimulated skin wrinkling in an ordinal fashion is doubtful.

## 1 Introduction

Small fiber neuropathy (SFN) is a form of peripheral neuropathy in which thinly myelinated Aδ-fibers and unmyelinated C-fibers are selectively affected without involvement of the large nerve fibers. The condition is clinically characterized by neuropathic pain and autonomic dysfunction (Lauria et al., 2012, Devigili et al., 2020, Sopacua et al., 2019). Diagnosis of SFN can be challenging since small nerve fibers are not always well investigated by electrophysiological testing and clinical presentation is difficult to interpret (Hoeijmakers et al., 2012).

Currently, SFN diagnosis is based on the following international criteria: (i) clinical signs of neuropathic pain and dysautonomia, (ii) a decreased intraepidermal never fiber density (IENFD) in skin biopsy and/or abnormal temperature threshold testing (TTT) and (iii) normal nerve conduction studies (NCS) (Tesfaye et al., 2010, Freeman et al., 2020, Devigili et al., 2019). In other words, a combination of a clinical, functional and structural approach to the diagnosis of SFN is reliable and relevant both for clinical practice and clinical trial design.

Although the diagnostic value of skin biopsy has been established (Lauria et al., 2010), it is an invasive method and assessment of obtained tissue is expensive and time consuming. Although the specificity of the IENFD is high, its sensitivity is moderate (De Greef et al., 2017, Fabry et al., 2020, Jin et al., 2018). While TTT is more accessible and non-invasive, the main disadvantage of this test is its moderate specificity (Maier et al., 2010, Shy et al., 2003). Also, test outcomes may be influenced by malingering or other nonorganic factors (Yarnitsky et al., 1994).

Previous studies have introduced Stimulated Skin Wrinkling (SSW) as a reliable and convenient tool to examine the sympathetic nerve function in hands and feet (Wilder-Smith, 2015, Falanga, 1993), in diabetic neuropathy (Ping Ng et al., 2013), and in HIV neuropathy (Mawuntu et al., 2018). Skin-wrinkling was originally induced by water immersion, which leads to diffusion and subsequent vasoconstrictive wrinkling of overlying skin (Wilder-Smith, 2004). Besides water, also EMLA (eutectic mixture of local anaesthetics) cream© was suggested as a usable vasoconstrictive factor. It is thought that EMLA causes vasoconstriction through direct effect on smooth muscle cells and postganglionic neuron Ca2+ channels (Wilder-Smith, 2004). Although SSW induced by water immersion and EMLA cream show nearly identical wrinkling, EMLA produces a more linear response curve than water and wrinkling persists for over 90 min allowing sufficient time for grading (Wilder-Smith and Chow, 2003).

In current clinical practice, skin wrinkling for assessing autonomic dysfunction in patients with SFN-like symptoms is performed in the hands and/or feet, using a published 5-point-scale (Vasudevan et al., 2000). This method of grading is subjective and the degree of natural wrinkles due to age and/or gender has been disregarded (Vasudevan et al., 2000). For example, skin elasticity, extensibility and echogenicity all decrease with age (Batisse et al., 2002). Considering the mentioned limitations in current SFN diagnostics, a more sensitive, specific and preferably non-invasive screening tool to detect small nerve fiber dysfunction would be of great value.

In the current study, we firstly aimed to obtain normative values for the SSW-test with EMLA, in a cohort of healthy individuals stratified for age under pre-defined standardized (room temperature, duration, assessed digits) assessment conditions. Assessment was done by the 5-point scale method. In addition, the inter-observer reliability was also examined as a minimum requirement for using the test in daily practice. Secondly, we have investigated whether the SSW assessment, using a new software program, the Digit Wrinkle Scan (DWS©), could improve the assessment of skin wrinkling in healthy individuals. The DWS© program digitally quantified the wrinkles in the fingertips, before and after EMLA application. For this analysis, the inter- and intra-observer reliability were determined.

## 2 Methods

We conducted a prospective cross-sectional diagnostic study in which we performed SSW with the 5-point scale method (part I) and the DWS© (part II) in healthy subjects.

Part I of the study took place between December 2012 and April 2013, while part II was performed between May 2017 and May 2018 at Maastricht University Medical Center+ (Maastricht UMC+), Maastricht, the Netherlands after having finished the development of the DWS© software. The study was approved by the local Medical Ethics Committee and written informed consent was obtained from each subject according to the declaration of Helsinki.

### 2.1 Subjects

Healthy subjects were recruited through advertisements in Maastricht UMC+. The following inclusion criteria applied: no SFN-related complaints as assessed using the SFN-Symptom Inventory Questionnaire (SFN-SIQ) and normal neurological testing (including Medical Research Council (MRC) grading, tendon reflexes and sensory testing). The SFN-SIQ evaluates several symptoms as well as autonomic symptoms in a simple manner. The questionnaire consists of 13 questions and was derived from the original SIQ, and from the composite autonomic symptoms scale (COMPASS) (Greco et al., 2017). Healthy subjects were excluded when they (i) had complaints of burning/tingling feet or hands; (ii) are known with previous neurological disorders, such as (poly)neuropathy, carpal tunnel syndrome, spinal cord and root disease, or significant limb trauma; (iii) have known conditions that may cause neuropathy: diabetes mellitus, hypothyroidism, renal failure, vitamin B12 deficiency, monoclonal gammopathy, alcohol abuse (more than five IU/day), malignancies, drugs that cause neuropathy (e.g. chemotherapy, amiodarone, propafenone); (iv) use skin cream at day of testing and (v) use antihypertensive drugs with effect on the sympathetic nervous system at peripheral effect (α1 adrenergic antagonists, β-blockers, calcium channel blockers, ACE inhibitors, AT1 receptor antagonists).

### 2.2 Study design

In order to create normative values for the SSW, participants were stratified for age, forming five age groups (20–29, 30–39, 40–49, 50–59, ≥ 60 years), each group consisting at least 5 males plus 5 females.

To determine the applicability of the DWS©, we randomly selected 35 new healthy participants.

### 2.3 Study procedure

All SSW examinations took place in a standardized, temperature controlled (21–24 °C) room, before lunch time. Subjects were instructed not to drink coffee or tea at least two hours before testing, since caffeine might influence autonomic function. In the first part, baseline photographs of the distal tip of the, 4th and 5th digit of both hands were taken, because it was reported that these fingers showed the clearest and most pronounced SSW with EMLA(Wilder-Smith and Chow, 2003). In the second part of the study, we took pictures of the distal tip of the 2nd, 3rd, 4th and 5th digit of both hands, since it is unknown if the DWS© would show other results during analyses. A review study has advised to use the average score of the 2nd, 3rd, 4th and 5th digit.(Wilder-Smith, 2015) However, in other studies the average of digit 3, 4 and 5, (Teohet al., 2008) or digit 2, 3, and 4 (Ping Ng et al., 2013) have been used. A fixed setup was used: the hand was held in front of a dark background with the palm of the hand towards the digital camera (CANON Eos 10D, macro lens). The camera was positioned at a standardized distance of 30 cm above the background with diffuse ring lighting around the lens.

In all subjects, skin wrinkling was induced by EMLA cream© (lidocaine 2.5% and prilocaine 2.5% AstraZeneca). Approximately 1 g (the amount needed to thickly and completely cover the distal digit pulp) of EMLA cream© was applied to the distal tip of the mentioned fingers of both hands and then left to soak into the skin for 30 min after covering with a Tegaderm© plaster. Any residual EMLA was removed afterwards. Subsequently, new photographs of EMLA-treated digits were taken in the same way as baseline pictures were made. All taken photographs were uploaded in a web-based program (MACRO), after they had been assigned a study number.

For the first part of the study, two trained observers graded the wrinkling pattern after SSW according to the previously published 5–point-scale (Vasudevan et al., 2000), in order to create normative values and to determine the inter-observer reliability of this assessment.

**Fig. 1:**
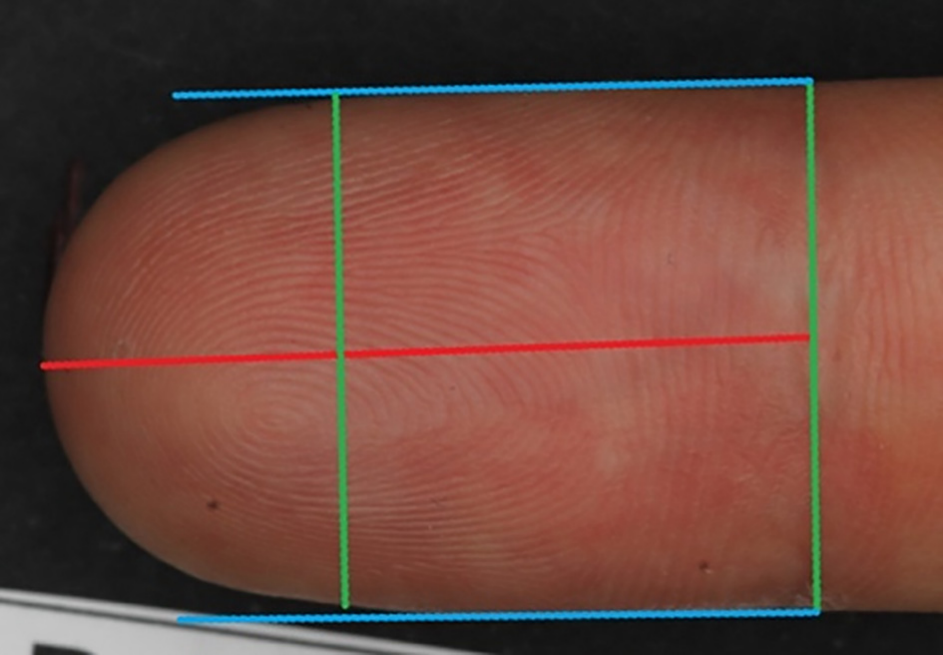
The length of the digit is determined by drawing the red straight line (in mm). The width is the mean of both green lines, which are drawn perpendicular to the blue lines (in mm). (For interpretation of the references to colour in this figure legend, the reader is referred to the web version of this article.)

**Fig. 2:**
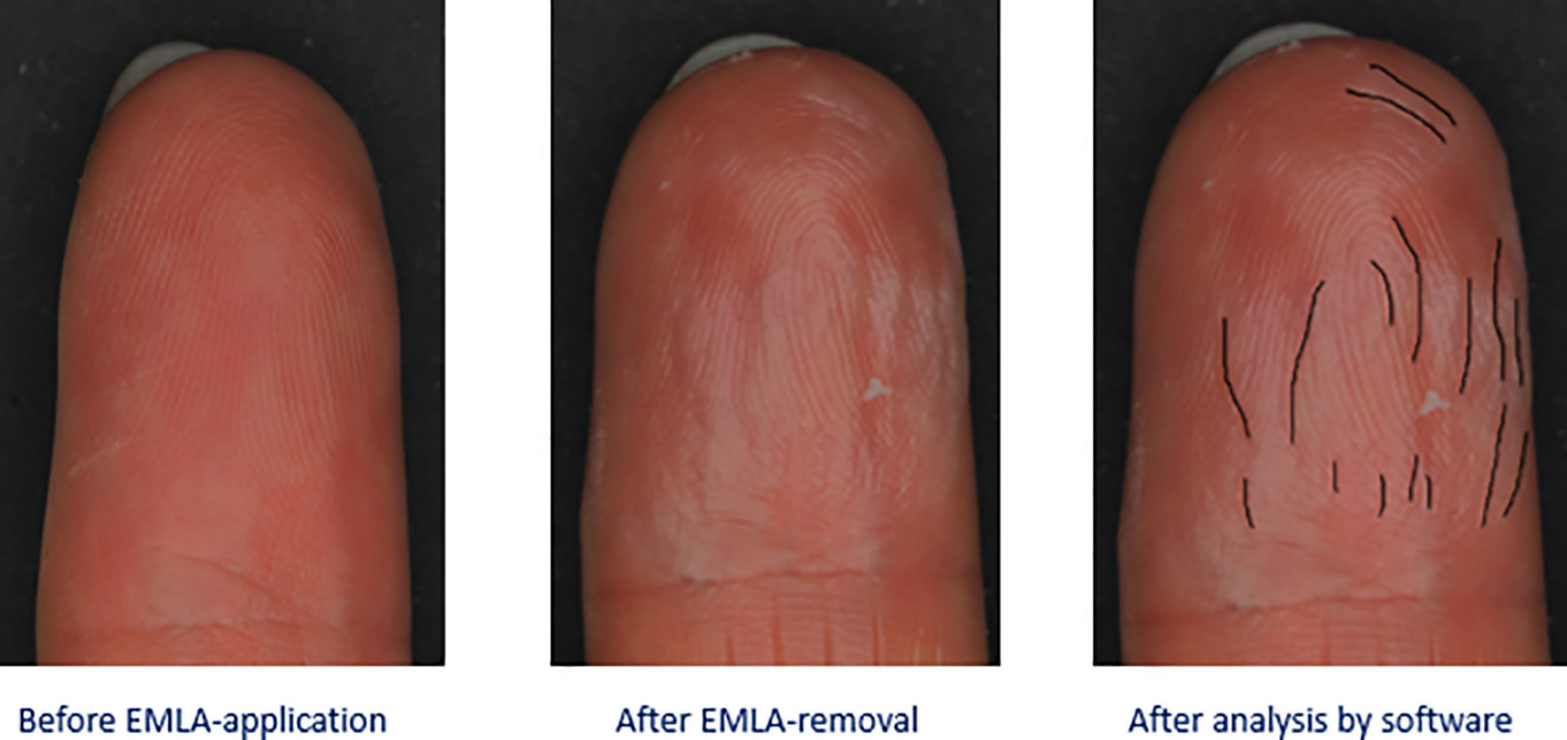
This is an example of the assessment process of one digit: a picture before EMLA application (left), after EMLA-removal (middle) and after uploading in the software and drawing the wrinkle lines (in mm) (right).

In the second part of the study, two independent observers carried out DWS© analyses in a blinded fashion. To determine the length and width of each distal digit tip, two straight lines were drawn after which the program automatically calculated the tip surface in millimeters^2^ (mm^2^) (Fig. 1). Subsequently, the researcher drew lines over all wrinkles (Fig. 2). Determination of surface area and total wrinkle length was done according to a protocol. The total length of wrinkles in millimeters (mm) and the total wrinkle length in mm/mm^2^ were calculated by the software. In order to determine the intra-observer reliability, one observer assessed each picture with an interval of, at least, two weeks without having access to the previous records.

### 2.4 Data analysis

Statistical analysis was carried out on a PC using Microsoft Excel and the IBM SPSS statistical package version 24.

The inter-observer variability for wrinkle scores according to the 5-point-scale was assessed using Cohen’s Kappa’s score. Furthermore, the inter-observer reliability was measured using intraclass correlation coefficient (ICC) for each digit by means of the Two-Way Random-Effects Model (Koo and Li, 2016). Lastly, the ICCs for the intra-observer reliability for the DWS© was calculated. The confidence interval was calculated with 95%.

The ICC values less than 0.5 were indicated as poor, values between 0.5 and 0.75 as moderate, values between 0.75 and 0.9 as good, and values greater than 0.90 as excellent reliability.

## 3 Results

### 3.1 5-point scale outcome measures

In part I, SSW was conducted in 82 healthy volunteers (women: *n* = 47, men: *n* = 35). Mean age for both gender was 49.8 (SD 16.9, range 21–77 years). Normative data per age group were shown for digit 4 and 5 in Table 1. With increasing age, the difference between wrinkling before and after SSW decreases. There was a minimal difference between digit 4 and 5.

**Table 1:** Normative values for SSW before and after EMLA application (range).

Age category (in years)	*t* = 0		*t* = 30	
	digit 4	digit 5	digit 4	digit 5
20–29	0	0	3.9 (3–4)	3.9 (3–4)
30–39	0	0	3.7 (3–4)	3.7 (1–4)
40–49	0.2 (0–1)	0.2 (0–1)	3.5 (2–4)	3.5 (1–4)
50–59	0	0	3.5 (1–4)	3.5 (1–4)
≥ 60	0.2 (0–3)	0.2 (2–3)	3.3 (1–4)	3.2 (1–4)

The inter-observer reliability scores at *t* = 0 were good for all examined digits (Table 2). The scores were moderate at *t* = 30 for digit 4 and 5; scores for digit 4 were slightly better than for digit 5 at *t* = 30.

**Table 2:** Inter-observer reliability (ICC) on *t* = 0 and *t* = 30.

*t* = 0	ICC	CI 95%
Digit 4, right	1.0	n/a
Digit 5, right	0.96	0.94–1.0
Digit 4, left	0.96	0.94–1.0
Digit 5, left	1.0	n/a
*t* = 30		
Digit 4, left	0.53	0.47–0.55
Digit 5, left	0.50	n/a

### 3.2 DWS outcome measures

In the second part of the study, we included a total of 35 healthy subjects (40% male; mean age 35.3 years; range 20–80 years). We calculated the ICC’s, based on the wrinkle length and the wrinkle length per mm^2^, at *t* = 0 and *t* = 30.

At *t* = 30 after EMLA-application, the inter-observer reliability was higher in the 4th and 5th digit, in comparison to the 2nd and 3rd digit (Table 3), but moderate for both. A good to excellent reliability (ICC ≥ 0.75) was only found on *t* = 30 in digit IV of the left hand (ICC: 0.788, CI: 0.570–0.895) for the wrinkle length/mm^2^ (Table 3).

**Table 3:** The inter-observer reliability of the line length and line length/mm2 before, and after EMLA-immersion.

	ICC (CI 95%) line length, *t* = 0	ICC (CI 95%) line length, *t* = 30	ICC (CI 95%) line length/mm^2^, *t* = 0	ICC (CI 95%) line length/mm^2^, *t* = 30
Digit, side				
2nd, right	0.656 (0.319–0.827)	0.608 (0.215–0.804)	0.464 (−0.062–0.279)	0.459 (−0.082–0.730)
3rd, right	0.520 (0.049–0.758)	0.368 (−0.265–0.684)	0.637 (0.280–0.817)	0.749 (0.498–0.875)
4th, right	0.658 (0.322–0.827)	0.674 (0.348–0.837)	0.535 (0.078–0.765)	0.686 (0.372–0.843)
5th, right	0.247 (−0.492–0.620)	0.765 (0.530–0.883)	0.560 (0.129–0.778)	0.711 (0.421–0.856)
2nd, left	0.548 (0.104–0.772)	0.370 (−0.262–0.685)	0.508 (0.026–0.752)	0.337 (−0.328–0.669)
3rd, left	0.642 (0.290–0.819)	0.632 (0.264–0.816)	0.650 (0.306–0.823)	0.226 (−0.550–0.613)
4th, left	0.333 (−0.332–0.663)	0.660 (0.311–0.832)	0.442 (−0.105–0.718)	0.788 (0.570–0.895)
5th, left	0.584 (0.177–0.190)	0.645 (0.281–0.825)	0.637 (0.281–0.817)	0.734 (0.462–0.869)

The intra-observer reliability (ICC) for the total group of 35 participants with the DWS showed a good reliability in two digits, namely 2nd digit (right) and 4th digit (left), 0.838 [CI: 0.578–0.937) and 0.827 [CI: 0.563–0.932] respectively after EMLA-application (Table 4). The remaining correlations were moderate.

**Table 4:** The intra-observer reliability of the line length and line length/mm^2^ before, and after EMLA-immersion.

	ICC (interval) line length/mm^2^	ICC (interval) *t* = 0 line length/mm^2^, *t* = 30
*Digit, side*		
2nd, right	0.374 (−0.530–0.760)	0.838 (0.578–0.937)
3rd, right	0.577 (−0.069–0.833)	0.585 (−0.078–0.804)
4th, right	0.469 (−0.341–0.798)	0.532 (−0.184–0.815)
5th, right	0.576 (−0.071–0.832)	0.698 (0.216−0.884)
2nd, left	0.416 (−0.475–0.769)	0.687 (0.209–0.876)
3rd, left	−0.118 (−1.826–0.557)	0.703 (0.248–0.882)
4th, left	0.600 (−0.011–0.842)	0.827 (0.563–0.932)
5th, left	0.692 (0.221–0.878)	0.628 (0.059–0.853)

## 4 Discussion

This study presents the normative values of SSW according to the 5-point scale assessment. Changes were found in SSW after EMLA application, namely a decrease with increasing age, suggesting a decrease of autonomic function. No effect was found for gender (results not published). Furthermore, reliability scores show moderate agreement. This suggests that the use of the categorical assessment, as intuitively simple as it might look and consequently the application of the normative values, is controversial and should be discouraged for clinical practice.

The age-dependent normative values that we obtained, showed a decrease in wrinkles with age. Various factors may contribute to age-related changes in the sensory system; a change in properties of the dermis, demyelination and fiber loss in peripheral nerves, and degenerative changes in the central nervous system (Wickremaratchi and Llewelyn, 2006). Although the extreme scores (i.e. score 0 or 4) can be well distinguished, the other categories are more doubtful. That could be the reason why relatively high ICCs were found before SSW and moderate agreement after EMLA-application. The scale used thus far is an ordinal one, based on classical test theory (CTT) (DeVellis, 2006). A major limitation of CTT is that scores create measurement at an ordinal level with unequal intervals that hamper accurate measurement of differences in scores and changes over time among individuals. Therefore, a more accurate and linear scale would be needed to properly assess skin wrinkling, both in terms of normative values as in clinical application, particularly when examining medical interventions.

For the second part of the study, we developed the DWS© in order to quantify the presence of natural wrinkling and wrinkling after EMLA application, on a ratio scale. DWS© provides a seemingly more specific, detailed and objective image of the presence of wrinkles on the digits as compared to the grading method. We found that the 4th and 5th finger of each hand after EMLA application had the highest ICC. These results may indicate that these fingers are best judged, which was previously reported (Wilder-Smith and Chow, 2003). However, the reliability scores cannot be marked as good/excellent and therefore hamper applicability in clinical practice.

The DWS© software program has several main advantages over the 5-point scale visual assessment. The DWS© takes physiological wrinkles into account, which are naturally present on the digit surface. In this way, the increase or decrease of wrinkles after EMLA-application can be determined. However, the DWS© protocol requires further improvement. Future studies should focus on improving the screening conditions (camera resolution, environmental light, picture angle), analysis techniques (3D analysis, Doppler imaging (Gechev, 2019)) and final outcome measures.

In conclusion, our results show that the SSW 5-point scale does not fulfill clinimetric requirements, which hampers its use in the clinical setting. Moreover, the DWS© is a more specific method to examine alterations in wrinkle formation after SSW, however, inter-observer reliability showed also moderate to good agreement at best. Therefore, more reliable techniques, in larger study samples, should be investigated (i.e. 3D-techniques, or subdermal visualization), to determine whether these methods could improve the assessments of autonomic dysfunction. If a reliable technique is available, international normative values, corrected for age, should be determined before it could serve as a tool in the diagnostics of autonomic dysfunction in neuropathies, like SFN.

## Author contributions

M.S, C.G, C.F, I.M and J.H designed the study. M.S, C.G and I.J. performed the measurements. M.S., C.G., I.J and B.G analysed the data. M.S, C.F, I.M and J.H wrote the paper with input from all authors.

## Conflict of interests

None of the authors have potential conflicts of interest to be disclosed.
